# The impact of physical therapy direct access policy on opioid shipments and opioid-related deaths: A difference-in-differences analysis

**DOI:** 10.1371/journal.pone.0333292

**Published:** 2026-06-10

**Authors:** Geronimo Bejarano, Peter Hull, James J. Young, David J. Meyers, Kevin N. Griffith

**Affiliations:** 1 Department of Health Services, Policy, and Practice, Brown University, Providence, Rhode Island, United States of America; 2 Department of Economics, Brown University, Providence, Rhode Island, United States of America; 3 National Bureau of Economic Research, Cambridge, Massachusetts, United States of America; 4 Schroeder Arthritis Institute, Krembil Research Institute, University Health Network, Toronto, Ontario, Canada; 5 Center for Muscle and Joint Health, Department of Sports Science and Clinical Biomechanics, University of Southern Denmark, Odense, Denmark; 6 Department of Health Policy, Vanderbilt University Medical Center, Nashville, Tennessee, United States of America; 7 Partnered Evidence-Based Policy Resource Center, Veterans Affairs Boston Healthcare System, Boston, Massachusetts, United States of America; University of Turin, ITALY

## Abstract

**Background:**

We exploit state-level policy changes for physical therapy direct access to estimate the effect of enacting direct access physical therapy policy on per-capita opioid pill volume (PCPV) and opioid-related deaths (ORD) compared to states without direct access to physical therapy.

**Methods:**

We used a staggered difference-in-differences analysis to assess changes in PCPV and ORD for states that did (treatment states) and did not enact (control states) direct access physical therapy policies between 2006–2014. Various sensitivity analyses including synthetic control methods were used to check the robustness of our results.

**Results:**

Treatment states had higher baseline PCPV (29.9 vs. 27.3) and ORD (27.2 vs. 16.9) compared to the control states. Pre-trends in PCPV and ORD were parallel between treatment and control states. After direct access physical therapy policies were enacted, PCPV was significantly reduced by −4.6 (95% confidence interval: −6.2 to −3.0) percentage points, a 15.4% relative reduction. There was no significant difference in ORDs (−2.3 [95% confidence interval: −7.2 to 2.6]).

**Conclusion:**

Direct access physical therapy policies significantly reduced PCPV but not ORDs. Our results suggest that administrative barriers to physical therapy may lead to greater opioid reliance and reduced population health.

## Introduction

The United States continues to have an ongoing public health crisis of opioid misuse and addiction with 17,000 prescription opioid-related deaths in 2021 and nearly 3 million people with opioid use disorder in 2020. [1–3] Musculoskeletal pain conditions are now the second leading cause of global disability, the most frequently cited reason to seek care and leading health care expenditure in the United States. [4–8] Opioids are most commonly prescribed in the treatment of musculoskeletal pain which unifies these two public health crises. [9,10]

For patients with a newly diagnosed musculoskeletal pain condition, 21% are prescribed opioids compared to only 10% prescribed physical therapy. [11] Increased utilization of safe and cost-effective alternative treatments is needed to address the opioid crisis, reduce unnecessary spending, and lower the disability burden of musculoskeletal pain. [12–14] Non-pharmacological treatments (e.g., exercise and manual therapy) commonly delivered by physical therapists are consistently recommended by clinical practice guidelines as first-line treatment options for all musculoskeletal pain conditions prior to initiation of opioid prescriptions. [15–19] Likewise, the Centers for Disease Control recommended prioritizing non-pharmacological treatments for pain because evidence suggests they are as effective as opioids with a lower risk of adverse events. [20]

If physical therapy for musculoskeletal pain does reduce opioid prescription volume, policymakers should consider removal of barriers to physical therapy access and encourage greater uptake of these services. Previous studies found an association between utilization of physical therapy for musculoskeletal pain and lower likelihood of opioid prescription. [21–23] However, these associations may be biased due to confounding from unobserved differences in the types and profiles of patient with musculoskeletal pain that seek care from physical therapists compared to those who seek care from medical doctors or other health care providers with prescription rights. Causal inference methods such as difference-in-differences (DID) allow us to derive causal estimates from policy change since they are not biased from unobserved time-invariant differences unlike regression and matching which have been used in previous studies. [24,25] DID methods are employed in other clinical areas to assess policies without bias from observed and unobserved time-varying confounders. [26]

To our knowledge, no previous study has analyzed the state level variation in physical therapy direct access laws using DID. Therefore, we exploit state-level physical therapy direct access policy variation over time to compare trends in opioid shipments to retail pharmacies between states that have and have not enacted direct access of physical therapy. We also analyzed opioid-related deaths as a secondary outcome and performed several sensitivity analyses to test the robustness of our findings to changes in our study design.

## Methods

### Data sources

We obtained data from a previous study that combined the 2006–2014 U.S. Drug Enforcement Administration’s Automation of Reports and Consolidated Orders System (ARCOS) pill shipment database and the Health Resources and Services Administration’s Area Health Resources Files (AHRF). [27–29] ARCOS contains detailed information on the distribution of controlled substances from manufacturers to retailers and was publicly released following a 2019 lawsuit by the Washington Post. AHRF contains county-level data on over 1000 variables including population demographics, healthcare infrastructure, health workforce, and health services utilization. We merged this dataset with information on state-level direct access physical therapy policy changes reported by the American Physical Therapy Association. [30] The results were reported in accordance with Strengthening the Reporting of Observational Studies in Epidemiology (STROBE) reporting guideline. [31]

### Physical therapy direct access policy

The exposure of interest was whether a state implemented a physical therapy direct access policy, applied to county-level outcome data within each state. Access differs between states as some require that a patient receives a referral from a physician prior to being able to receive care from a physical therapist. Several states enacted policies allowing patients direct access to physical therapy without a referral during our study period. In 2010, Hawaii passed physical therapy direct access followed by Kansas, California, and Indiana in 2013 therefore these states are our treated states. As of 2014, Alabama, Illinois, Louisiana, Mississippi, Missouri, New Mexico, Oklahoma, and Texas still did not have physical therapy direct access therefore these states served as the never treated control states. Any other states were excluded due to having partial or provisional direct access prior to the study periods.

### Outcomes

Our primary outcome was the per capita pill volume (PCPV) of opioids, defined as the annual per-capita shipments of oxycodone and hydrocodone pill shipment to retail pharmacies, calculated at the county-level in the ARCOS database. Our secondary outcome was the annual age-adjusted opioid-related death rate per 100,000 population at the county-level as reported by the Centers for Disease Control. [28]

### Statistical analysis

We conducted a DID analysis to compare differences in trends of PCPV before and after state-level physical therapy direct access policy changes using county and year fixed effects, where the estimate of interest is the interaction term between county and year fixed effects. DID analysis enables us to estimate the causal effect of policy changes on the treated states under the assumption their pre-trends would have continued parallel to the control group had the policy not been enacted. [24]

Our primary analysis used a staggered DID estimator allowing us to account for differential timing (2010 and 2013) of physical therapy direct access policy changes. [25] We used four pre-periods and two post-periods in the analysis since there are only two post periods for the 2013 policy change. Standard errors were clustered at the county level in the primary analysis, the year before treatment served as the baseline, and regressions were weighted by state population. [32] In DID analyses, adjustment for covariates is used to relax the parallel trends assumption. [24] This differs from randomized controlled trials where covariate adjustment is used to increase precision of the estimate or observational regression studies where covariate adjustment is used to attempt to control for confounding. [33,34] Since our pre-trends appeared to be parallel based on event study plots, we consider the unadjusted model as the primary analysis. We conducted a DID analysis adjusting for continuous variables including age, income, uninsurance rates, and dual eligibility rates and another DID analysis with standard errors clustered at the state-level as sensitivity analyses. Our secondary analysis used the same methods with opioid related deaths as the outcome.

To further check the robustness of our results, we conducted a synthetic control analysis to compare Hawaii (policy changed in 2010) to a synthetic Hawaii created using weights derived from the never treated states. [35] Synthetic control is more appropriate with one exposed state therefore, we used Hawaii as the treatment group since it is the only state to have changed policy in 2010. This method assigns weights to each control state; weights are determined via a data-driven optimization algorithm (root-mean square prediction error) to minimize pre-period differences in outcomes between treated and control states. The weighted sum of control states is then referred to as a synthetic control (hereafter “synthetic Hawaii”). All statistical analyses were conducted using Stata, version 18.0. The data is at the county-level and without any ability to identify individuals and was accessed for research purposes on February 19, 2024.

## Results

The treatment group consisted of 2,268 counties which had a plurality aged 46–64 years (28.0%), were split on gender (50.0% female), and were majority White (91.0%) (Table 1). The control group consisted of 7,555 counties with a plurality aged 45–64 years (27.6%) and a majority that were female (50.4%) and White (82.9%). There was a greater proportion of dual-eligible (12.6% vs. 11.2%), uninsured individuals under 65 years old (20.0% vs. 16.3%), and lower per-capita income ($32,521 vs. $37,372) in the control group than the treatment group. Baseline PCPV (29.9 vs. 27.3) and ORD (27.2 vs. 16.9) was higher in the treatment group than the control group.

**Table 1:** Baseline Characteristics of Treatment and Control States.

Characteristics		Treatment	Control
Number of Counties	No. (%)	2,268 (23.1)	7,555 (76.9)
Age (%)	25-44 years	23.8%	24.1%
	45-64 years	28.0%	27.6%
	65 + years	15.9%	15.8%
Sex (%)	Male	49.96%	49.76%
Race (%)	Asian	2.7%	0.8%
	Black	2.6%	12.5%
	White	91.0%	82.9%
	Other	3.7%	3.8%
Ethnicity (%)	Hispanic	11.2%	12.6%
Insurance Status (%)	Dual-Eligible	3.2%	4.4%
	Uninsured under 65	16.3%	20.0%
Per-capita income ($)	Mean (SD)	$37,372 ($10,287)	$32,520.70 ($7,712)
Opioid PCPV	Mean (SD)	29.9 (14.7)	27.3 (14.7)
Opioid-related deaths (per 100,000)	Mean (SD)	27.2 (79.2)	16.9 (59.3)

### Changes in opioid prescription volume

**Fig 1:**
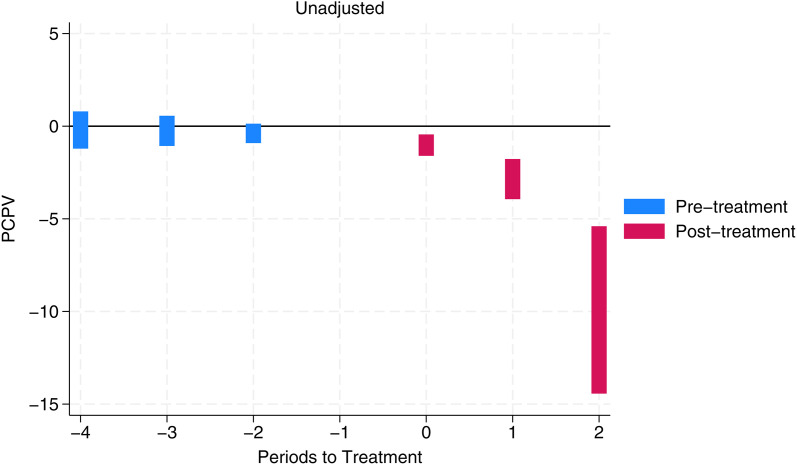
Unadjusted staggered difference-in-differences estimates of the average treatment effect of physical therapy direct access policy on the PCPV of the treated states. Notes: Difference-in-Difference models were estimated using CSDID package in STATA. Dots represent the point estimate and shaded bars represent the 95% confidence intervals. Since physical therapy direct access policy was implemented in different years, the periods to treatment represent different years depending on the treated state. All estimates are derived using the period before baseline to improve interpretation of the figure. PCPV is per-capita per volume of opioid pill shipments. Standard errors clustered at the county level.

**Fig 2:**
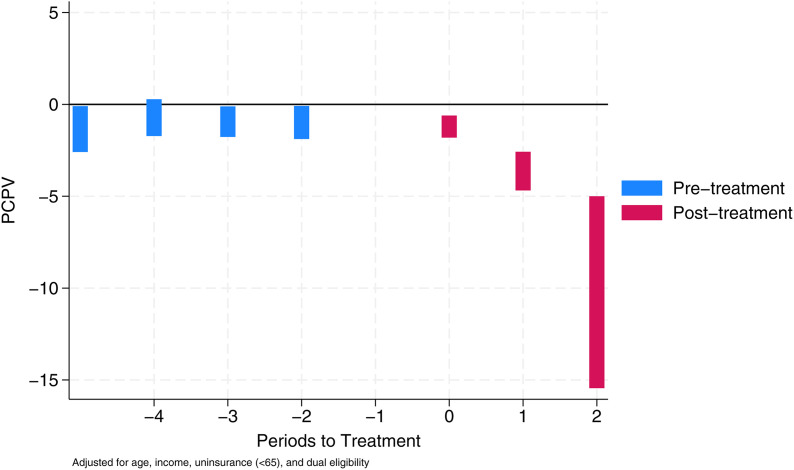
Adjusted staggered difference-in-differences estimates of the average treatment effect of physical therapy direct access policy on the PCPV of the treated states. Notes: Difference-in-Difference models were estimated using CSDID package in STATA. Dots represent the point estimate and shaded bars represent the 95% confidence intervals. Since physical therapy direct access policy was implemented in different years, the periods to treatment represent different years depending on the treated state. All estimates are derived using the period before baseline to improve interpretation of the figure. PCPV is per-capita per volume of opioid pill shipments. Standard errors clustered at the county level.

Prior to the direct access physical therapy policy change, trends in PCPV were similar in the treatment group compared to the control group. (Fig 1 and Fig 2) In the DID analysis, we found that PCPV significantly decreased by −4.6 (−6.2 to −3.0) percentage points after the direct access physical therapy policy change, which is a 15.4% relative reduction compared to baseline. (Table 2) Our sensitivity analyses including covariate-adjusted DID models, state-level clustering models, and synthetic control analyses for Hawaii were consistent with primary analysis results showing a reduction in PCPV after change in physical therapy direct access. (S1 and S2 Tables, S1 and S2 Figures in S1 File)

**Table 2:** Staggered Difference-in-Differences estimates of direct access to physical therapy on PCPV and ORD of treated states.

	PCPV		ORD	
	Unadjusted	Adjusted	Unadjusted	Adjusted
ATT (95% CI)	−4.6 (−6.2 to −3.0)	−5.02 (−6.85 to −3.19)	−2.3 (−7.2 to 2.6)	−8.81 (−20.42 to 2.79)
P-Values	<0.001	<0.001	0.4	0.137

Notes: ATT: Average treatment on the treated, PCPV: per-capita per volume of opioid pill shipments, ORD: opioid-related deaths. Difference-in-Difference models were estimated using CSDID package in STATA. Standard errors clustered at the county level. Adjusted estimates were adjusted for age, income, uninsurance, and dual eligibility.

### ORD outcome

**Fig 3:**
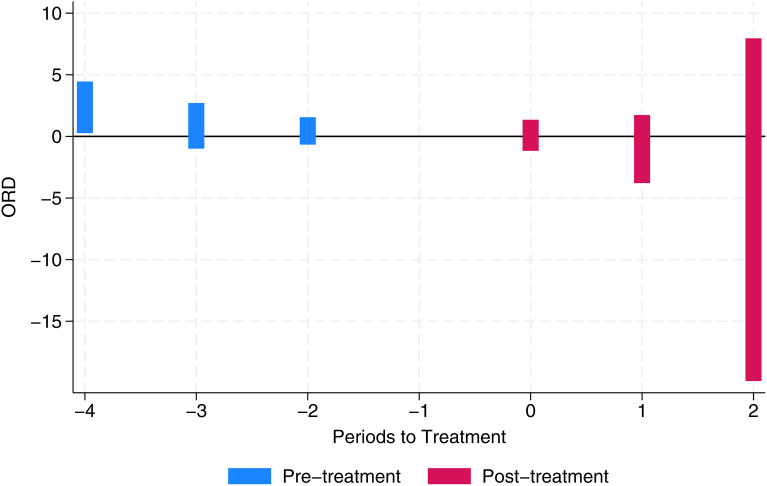
Unadjusted and adjusted staggered Difference-in-Differences estimates of the average treatment effect of physical therapy direct access policy on the ORD of the treated states. Notes: Difference-in-Difference models were estimated using CSDID package in STATA. Dots represent the point estimate and shaded bars represent the 95% confidence intervals. Since physical therapy direct access policy was implemented in different years, the periods to treatment represent different years depending on the treated state. All estimates are derived using the period before baseline to improve interpretation of the figure. ORD is opioid related deaths. Standard errors clustered at the county level.

**Fig 4:**
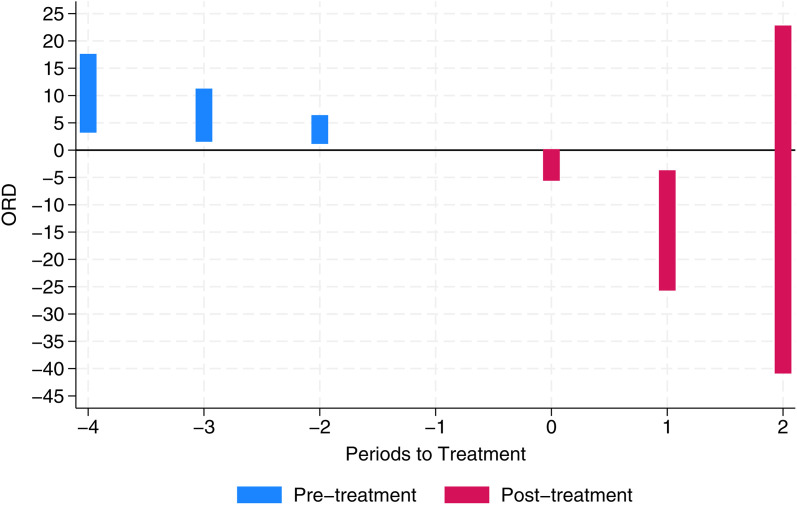
Adjusted staggered Difference-in-Differences estimates of the average treatment effect of physical therapy direct access policy on the ORD of the treated states. Notes: Difference-in-Difference models were estimated using CSDID package in STATA. Dots represent the point estimate and shaded bars represent the 95% confidence intervals. Since physical therapy direct access policy was implemented in different years, the periods to treatment represent different years depending on the treated state. All estimates are derived using the period before baseline to improve interpretation of the figure. ORD is opioid related deaths. Standard errors clustered at the county level.

Prior to direct access physical therapy policies, trends in ORDs were similar between treatment and control states. (Fig 3 and Fig 4) We found no statistically significant difference in ORDs (−2.3, 95% CI: −7.2 to 2.6) between treated and control states after enacting direct access physical therapy policies.

## Discussion

Reduced use of opioids is imperative in order to diminish the risks of addiction while providing guideline concordant care for musculoskeletal pain. [36] Our findings provide the first estimates using rigorous causal inference methods of the effect of direct access physical therapy policies on opioid use and mortality. We find that direct access to physical therapy significantly reduced PCPV but did not reduce ORDs.

Several previous studies assessed the effect of direct access to physical therapy, however, the relevance of their findings for policymaking may be limited by selection bias due to unobserved confounders. [37,38] For example, direct access was associated with less visits, reductions in diagnostic imaging, and lower healthcare costs for low back pain compared to states without direct access to physical therapy. [39] However, the authors acknowledge that since they used logistic regression to compare differences between those with and without direct access to physical therapy, they were only able to establish association and not causation due to possible bias from unobserved confounders even after their adjustments (e.g., attitudes towards opioids where those who seek treatment from physical therapists would deny opioids even if they saw a medical doctor). By using DID methods, our findings add to the literature by providing rigorous quasi-experimental evidence on the effects of direct access physical therapy policies while accounting for both observed and time-invariant unobserved confounders.

Increasing direct access to physical therapy can play a pivotal role in addressing both the opioid and musculoskeletal pain public health crises. Our findings strengthen the evidence indicating beneficial effects of physical therapists as first-line care providers to improve patient and public health. [13,14,40] As of 2024, only two states (Alabama and Mississippi) do not have direct access to physical therapy and rank amongst the highest for opioid dispensing rates and PCPV. [27,41] Our findings along with the current literature support consideration for direct access physical therapy policies to potentially reduce the burden of opioids. [14,42] Barriers remain for patients to access physical therapy beyond direct access policies including lack of insurance coverage for non-pharmacological care, limited number of physical therapists in their insurance networks, co-pay amounts greater for physical therapy than medical doctors, and lack of knowledge about the benefits and low risk of non-pharmacological compared to pharmacological care. [43,44]

### Limitations

This is the first study to use a staggered DID method to assess direct access physical therapy policy and to our knowledge in musculoskeletal pain more broadly. Our findings were robust to several different sensitivity analyses including using a different novel causal inference methodology synthetic control that has been used to assess policy change in other health conditions. [45] Given Hawaii’s unique demographic characteristics, the synthetic control estimates provide the effect of physical therapy direct access policies for Hawaii which may not be generalizable to other states. However, given the alignment of the synthetic control results with the DID analyses, it provides further validity of the robustness of our findings. Our study design is observational and we cannot rule out that outcome trends would have diverged between treated and control states in the absence of policy changes. However, our pre-trends appear to be parallel between treatment and control states which strengthens our belief that the parallel trends assumption holds in our analysis. [24] While the DID analysis with county and year fixed effects helps account for common temporal shocks, differential adoption of other opioid related policies (e.g., pill mill laws and prescribing limits) may confound our estimates. Additionally, the treatment states have higher PCPV and ORD at baseline than control groups, which could bias the DID analysis if trends differed. However, trends between treated and control states during the periods before treatment were parallel suggesting that they would continue parallel if it had not been for the physical therapy direct access policy change. Another limitation is the outcome of PCPV may not be generalizable to opioid prescriptions, real-world opioid utilization or misuse. Our data also does not distinguish between appropriate and inappropriate opioid prescriptions or physical therapy use, therefore our PCPV findings are limited in that they cannot be interpreted as reducing inappropriate opioid prescribing behavior. Since our data do not include opioid prescription volume or use, future research is needed to see if these results translate to opioid prescriptions or use and other low value treatments for musculoskeletal pain (e.g., surgery or injections). Mortality rates can require large sample size to properly power analyses to identify statistically significant effects due to their rarity. ORD outcomes in our analysis have 95% confidence intervals that cannot rule out that the null effect is due to low power and not a lack of effect of physical therapy direct access policy on ORD. Our analysis does not account for cross-state spillovers, which could bias treatment effects toward the null if patients in control states use services in neighboring treated states or vice versa. Finally, physical therapy direct access policies can be heterogenous (e.g., amount of physical therapy visits restricted) which may limit the generalizability of our findings.

## Conclusion

In this study, direct access physical therapy policies significantly reduced PCPV. This is the first study to use a difference-in-differences method to estimate the effects of direct access physical therapy policy, confirming previous studies that have provided correlational estimates. Our findings highlight the importance of access to physical therapy as a key contributor to improving opioid and musculoskeletal pain public health crises.

## Supporting information

S1 File The Impact of Physical Therapy Direct Access Policy on Opioid Shipments: A Difference-in-Differences Analysis. (DOCX)
