# Supplementary material for: The impact of physical therapy direct access policy on opioid shipments and opioid-related deaths: A difference-in-differences analysis
Source: PLoS One. 2026 Jun 10;21(6):e0333292. doi: 10.1371/journal.pone.0333292 (PMC13252735; doi:10.1371/journal.pone.0333292)
Supplement: S1 File — (DOCX) [file pone.0333292.s001.docx]

**Supporting Information for: The Impact of Physical Therapy Direct Access Policy on Opioid Shipments: A Difference-in-Differences Analysis**

**S1 Figure:** Staggered Difference-in-Differences estimates of the average treatment effect of physical therapy direct access policy on the PCPV of the treated states using standard errors clustered at the state-level


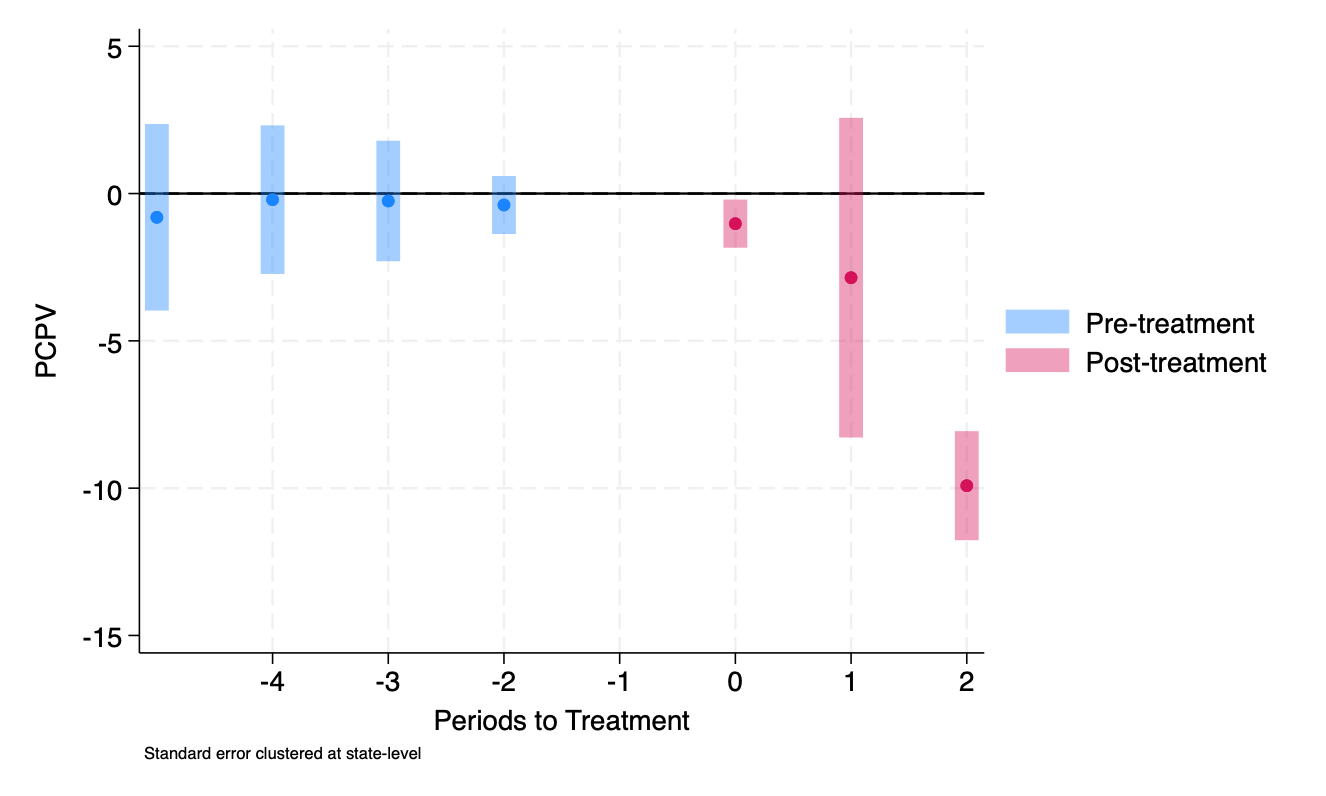


**S2 Figure:** Synthetic control estimates comparing Hawaii to a synthetic Hawaii


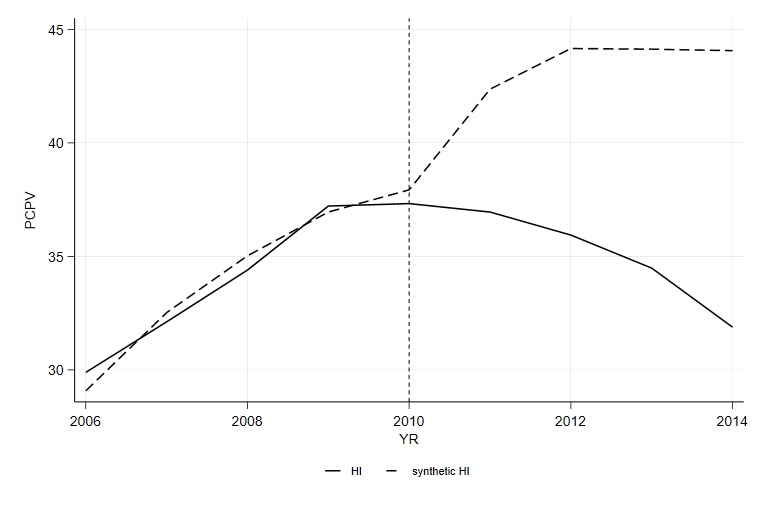


**Notes:** Synth and synth_runner packages were used in Stata. Synthetic Hawaii was created using weights from the states without direct access physical therapy policies derived from synth package.

**S1 Table:** Synthetic control weights used to create synthetic Hawaii

| **State** | **Unit Weight** |
| --- | --- |
| AL | 0 |
| IL | 0.2 |
| LA | 0.7 |
| MI | 0.1 |
| MO | 0 |
| MS | 0 |
| NM | 0 |
| OK | 0 |
| TX | 0 |

**S2 Table** Synthetic control estimates of physical therapy direct access on PCPV comparing Hawaii to synthetic Hawaii

|  | **PCPV** | | | |
| --- | --- | --- | --- | --- |
|  | 1-Year Post | 2-Year Post | 3-Year Post | 4-Year Post |
| ATT | -5.4 | -8.2 | -9.6 | -12.2 |
| Standardized P-Values | 0.2 | 0.1 | 0.3 | 0.1 |

**Notes:** Synth and synth_runner packages were used in Stata. Synthetic Hawaii was created using weights from the states without direct access physical therapy policies derived from synth package. Standardized p-values are the probability that this estimate would happen by chance.
