# Supplementary material for: The impact of physical therapy direct access policy on opioid shipments and opioid-related deaths: A difference-in-differences analysis
Source: PLoS One. 2026 Jun 10;21(6):e0333292. doi: 10.1371/journal.pone.0333292 (PMC13252735; doi:10.1371/journal.pone.0333292)
Supplement: Attachment — Submitted filename: Report-PONE-D-25-49217.docx [file pone.0333292.s002.docx]

**The Impact of Physical Therapy Direct Access Policy on Opioid Shipments and Opioid-related Deaths: A Difference-in-Differences Analysis**

**(Review Suggestions for further Improvement)**

**Abstract**

It is only wise that it expressly states that the policy exposure is measured at the state level, whereas the outcomes are measured at the county level. More so, a brief definition of the term per-capita volume of opioid pills in the form of annual per-capita shipments of oxycodone and hydrocodone pills to retail pharmacies should be given.

**Introduction**

The introduction of the knowledge gap can be made sharper; that is, one should add one or two sentences to the Introduction that will clearly indicate that no previous study has exploited inter-state variation in direct access physical therapy laws to measure the causal impact of the latter on the volumes of opioid pills shipped and opioid-related mortality.

**Methods** (**Data sources & exposure)**

1. The analysis clearly indicates that the treated and control states of county-year observations are clustered, with standard errors being clustered at the county level; sensitivity analyses are also done by clustering at the state level.
2. The selection of only Hawaii, Kansas, California, and Indiana as treated states was based on each one of them implementing the policy of direct-access in the study period and on similar demographic and economic factors; the eight never-treated states were chosen as a control group to achieve matching on these factors, and any states that implemented a partial access policy, or whose timing did not coincide, or which had incomplete data were excluded.
3. A brief description is provided of the heterogeneity of direct-access policies, which include free access to the policy, procedures with visitor time restrictions, etc.; this diversity can affect the scale of the estimated impacts and constrain the extrapolation of the policy findings.

**Outcomes**

ORD outcome is measured in 100,000 inhabitants per 100,000 inhabitants, but these values are not directly indicated in the manuscript as being age-adjusted or unadjusted. The latter methodological ambiguity should be tackled because the age distributions of different counties may differ significantly, thus affecting the comparability of the reported rates.

**Statistical analysis**

1. The Difference-in-Differences (DiD) model specification could be improved in terms of increased transparency, specifically in the clear definition of fixed-effects structures (e.g., state x year versus county x year) and the clear description of how time-varying covariates (including more than the usual controls of age, income, uninsurance, and dual eligibility) are operationalized (continuous or categorical).
2. A limitation of the study is that cross-state spillovers (like cross-border prescribing or mortality) may be present in the study. In addition, the synthetic-control approach is overly brevily treated, with more information given about how the pre-policy fit is assessed (as e.g. by root-mean-square prediction error), the use of either placebo-tests or permutation tests, and how the temporal window in which the synthetic control has been constructed and used is to be specified.

**Results**

1. The differences in the baselines in the treatment and control states, including high PCPV and ORD value, differences in income and coverage of insurance, are accepted but not discussed in terms of the possible impact on cause interpretation or external validity. It would therefore be wise to give a brief evaluation on whether the treated states are highly-burdened contexts.
2. The results are typically presented clearly, but the specifics that are of interest are only located in the appendix (e.g., synthetic control results, sensitivity analysis). In the primary Results section, it would be a good idea to summarise how these sensitivity estimates match (i.e. concordant or divergent) the major findings of DID.
3. In the case of ORD, the specified non-significant effect with the wide confidence interval that includes both substantive decreases and increases is reported, yet not placed in a relative change context or in a statistical power setting.

**Discussion**

1. The null effect of opioid-related mortality should be described in greater detail: the shortness of the follow-up, the research involving a high number of other mortality drivers, and the potential lack of statistical power in the form of county-level mortality results should be mentioned.
2. The impediments to PT access and the suggestion that the remaining states should emulate direct-access policies should be presented with more caution, clearly identifying those findings that are directly elucidated by this analysis as opposed to the conclusions made by the rest of the literature and the historical background of such policies.

**Limitations (Additional limitations to consider)**

The possibility of discrepancies between the distribution of the tablets with opioids and the trend of the real usage or intake of the prescription is real. In addition, the present study fails to involve data related to non-prescribed opioid use or poly-substance abuse.

**References**

It is seen that some of the web-based sources and Google Scholar entries, including the Washington Post database of ARCOS and the AHRF, are currently quoted by using only the path scholar.google.com and not by mentioning original reports or technical documents. To be on the safe side and assure verifiability in the long term, it would be wise to include direct quotes of the main sources.
