# Supplementary material for: The impact of physical therapy direct access policy on opioid shipments and opioid-related deaths: A difference-in-differences analysis
Source: PLoS One. 2026 Jun 10;21(6):e0333292. doi: 10.1371/journal.pone.0333292 (PMC13252735; doi:10.1371/journal.pone.0333292)
Supplement: Attachment — Submitted filename: PLOS ONE Response Letter 2026 03 14.docx [file pone.0333292.s004.docx]

March 14^th^, 2026

Dear Dr. Baratta,

Thank you for the opportunity to revise and resubmit our manuscript, “The Impact of Physical Therapy Direct Access Policy on Opioid Shipments and Opioid-related Deaths: A Difference-in-Differences Analysis” for consideration at *PLOS One*. The reviewer’s and editor’s comments have been very helpful and strengthened the paper. Our responses are below.

Sincerely,

Kevin N. Griffith, Ph.D.

Assistant Professor

Department of Health Policy

Vanderbilt University School of Medicine

**Reviewer #1**

| Reviewer Comments | Author Reply |
| --- | --- |
| It is only wise that it expressly states that the policy exposure is measured at the state level, whereas the outcomes are measured at the county level. More so, a brief definition of the term per-capita volume of opioid pills in the form of annual per-capita shipments of oxycodone and hydrocodone pills to retail pharmacies should be given. | We thank the reviewer for the helpful suggestion. We have added in the methods: “The exposure of interest was whether a state implemented a physical therapy direct access policy, applied to county-level outcome data within each state.” As well as “Our primary outcome was the per capita pill volume (PCPV) of opioids, defined as the annual per-capita shipments of oxycodone and hydrocodone pill shipment to retail pharmacies, calculated at the county-level in the ARCOS database” |
| The introduction of the knowledge gap can be made sharper; that is, one should add one or two sentences to the Introduction that will clearly indicate that no previous study has exploited inter-state variation in direct access physical therapy laws to measure the causal impact of the latter on the volumes of opioid pills shipped and opioid-related mortality. | As suggested by the reviewer, we have added to the introduction with: “To our knowledge, no previous study has used the state level variation in physical therapy direct access laws using DID. Therefore, we exploit state-level physical therapy direct access policy variation over time to compare trends in opioid shipments to retail pharmacies between states that have and have not enacted direct access of physical therapy.” |
| The analysis clearly indicates that the treated and control states of county-year observations are clustered, with standard errors being clustered at the county level; sensitivity analyses are also done by clustering at the state level. | We have clarified the analysis as suggested by the reviewer with: “Standard errors were clustered at the county level in the primary analysis using the year before treatment as the baseline and regressions were weighted by state population” and “DID analysis with standard errors clustered at the state-level as sensitivity analyses” |
| The selection of only Hawaii, Kansas, California, and Indiana as treated states was based on each one of them implementing the policy of direct-access in the study period and on similar demographic and economic factors; the eight never-treated states were chosen as a control group to achieve matching on these factors, and any states that implemented a partial access policy, or whose timing did not coincide, or which had incomplete data were excluded. | We have clarified our methods as suggested by the reviewer with: “Any other states were excluded due to having partial or provisional direct access prior to the study periods.” |
| A brief description is provided of the heterogeneity of direct-access policies, which include free access to the policy, procedures with visitor time restrictions, etc.; this diversity can affect the scale of the estimated impacts and constrain the extrapolation of the policy findings | As the reviewer suggested, we have added to the limitations section with: “Finally, physical therapy direct access policies can be heterogenous (e.g., amount of physical therapy visits restricted) which may limit the generalizability of our findings.” |
| ORD outcome is measured per 100,000 inhabitants, but these values are not directly indicated in the manuscript as being age-adjusted or unadjusted. The latter methodological ambiguity should be tackled because the age distributions of different counties may differ significantly, thus affecting the comparability of the reported rates | We have clarified in our methods that these are age-adjusted outcomes with: “Our secondary outcome was the annual age-adjusted opioid-related death rate per 100,000 population at the county-level as reported by the Centers for Disease Control.” |
| The Difference-in-Differences (DiD) model specification could be improved in terms of increased transparency, specifically in the clear definition of fixed-effects structures (e.g., state x year versus county x year) and the clear description of how time-varying covariates (including more than the usual controls of age, income, uninsurance, and dual eligibility) are operationalized (continuous or categorical). | We improved the specification of the DID model in the methods with: “We conducted a DID analysis to compare differences in trends of PCPV before and after state-level physical therapy direct access policy changes using county and year fixed effects, where the estimate of interest is the interaction term between county and year fixed effects.” And “We conducted a DID analysis adjusting for continuous variables including age, income, uninsurance rates, and dual eligibility rates” |
| A limitation of the study is that cross-state spillovers (like cross-border prescribing or mortality) may be present in the study. In addition, the synthetic-control approach is overly brevily treated, with more information given about how the pre-policy fit is assessed (as e.g. by root-mean-square prediction error), the use of either placebo-tests or permutation tests, and how the temporal window in which the synthetic control has been constructed and used is to be specified. | In the limitation section, we have addressed the potential for cross-state spillovers with: “Our analysis does not account for cross-state spillovers, which could bias treatment effects toward the null if patients in control states use services in neighboring treated states or vice versa.”  For the synthetic control method, we have expanded the methods with: “This method assigns weights to each control state; weights are determined via a data-driven optimization algorithm (root-mean square prediction error) to minimize pre-period differences in outcomes between treated and control states.” |
| The differences in the baselines in the treatment and control states, including high PCPV and ORD value, differences in income and coverage of insurance, are accepted but not discussed in terms of the possible impact on cause interpretation or external validity. It would therefore be wise to give a brief evaluation on whether the treated states are highly-burdened contexts | We have added in the limitations section with: “Additionally, the treatment states have higher PCPV and ORD at baseline than control groups, which could bias the DID analysis if trends differed. However, trends between treated and control states during the periods before treatment were parallel suggesting that they would continue parallel if it had not been for the physical therapy direct access policy change.” |
| The results are typically presented clearly, but the specifics that are of interest are only located in the appendix (e.g., synthetic control results, sensitivity analysis). In the primary Results section, it would be a good idea to summarise how these sensitivity estimates match (i.e. concordant or divergent) the major findings of DID. | The reviewer makes a good suggestion to further clarify the key sensitivity findings that align with the main results. We have added this in the results with: “Our sensitivity analyses including covariate-adjusted DID models, state-level clustering models, and synthetic control analyses for Hawaii were consistent with primary analysis results showing a reduction in PCPV after change in physical therapy direct access.” |
| In the case of ORD, the specified non-significant effect with the wide confidence interval that includes both substantive decreases and increases is reported, yet not placed in a relative change context or in a statistical power setting. The null effect of opioid-related mortality should be described in greater detail: the shortness of the follow-up, the research involving a high number of other mortality drivers, and the potential lack of statistical power in the form of county-level mortality results should be mentioned. | We agree that ORD and mortality rates more broadly can be underpowered and thus have statistical uncertainty. We have added this to our limitations with: “Mortality rates can require large sample size to properly power analyses to identify statistically significant effects due to their rarity. ORD outcomes in our analysis have 95% confidence intervals that cannot rule out that the null effect is due to low power and not a lack of effect of physical therapy direct access policy on ORD.” |
| The impediments to PT access and the suggestion that the remaining states should emulate direct-access policies should be presented with more caution, clearly identifying those findings that are directly elucidated by this analysis as opposed to the conclusions made by the rest of the literature and the historical background of such policies | We have presented our policy implications in the discussion with more caution, including removing “should” and softer wording with: “Our findings along with the current literature support consideration for direct access physical therapy policies to potentially reduce the burden of opioids.(14,43) Barriers remain for patients to access physical therapy beyond direct access policies including lack of insurance coverage for non-pharmacological care, limited number of physical therapists in their insurance networks, co-pay amounts greater for physical therapy than medical doctors, and lack of knowledge about the benefits and low risk of non-pharmacological compared to pharmacological care.(44,45)” |
| The possibility of discrepancies between the distribution of the tablets with opioids and the trend of the real usage or intake of the prescription is real. In addition, the present study fails to involve data related to non-prescribed opioid use or poly-substance abuse. | We agree with the reviewer that opioid shipments may not reflect real-world utilization. We do not have access to claims data to study this outcome and therefore describe this in the limitations and suggestion for future studies with: “Another limitation is the outcome of PCPV may not be generalizable to opioid prescriptions, real-world opioid utilization or misuse. Since our data do not include opioid prescription volume or use, future research is needed to see if these results translate to opioid prescriptions or use and other low value treatments for musculoskeletal pain (e.g., surgery or injections).” |
| It is seen that some of the web-based sources and Google Scholar entries, including the Washington Post database of ARCOS and the AHRF, are currently quoted by using only the path [scholar.google.com](http://scholar.google.com) and not by mentioning original reports or technical documents. To be on the safe side and assure verifiability in the long term, it would be wise to include direct quotes of the main sources. | We have removed the Google Scholar entry of the Washington Post database and included citations for several studies that provide the Arcos database including:  27. Griffith KN, Feyman Y, Auty SG, Crable EL, Levengood TW. Implications of county-level variation in U.S. opioid distribution. *Drug Alcohol Depend*. 2021;219:108501. doi:10.1016/j.drugalcdep.2020.108501  28. Griffith KN, Feyman Y, Auty SG, Crable EL, Levengood TW. County-level data on U.S. opioid distributions, demographics, healthcare supply, and healthcare access. *Data Brief*. 2021;35:106779. doi:10.1016/j.dib.2021.106779  29. Amico A, Verginer L, Schweitzer F. Tracing Opioids Across the US: A High-Resolution Pharmaceutical Distribution Dataset. *Sci Data*. 2024;11(1):774. doi:10.1038/s41597-024-03534-3 |

**Reviewer #2**

| Reviewer Comment | Author Reply |
| --- | --- |
| A good study has been conducted to control the use of opioids, but due to the differences in the need for opioids in different types of diseases, the need for physical therapy, the existence of specific surgeries, etc., it has not been considered. As a result, this comparison, in my opinion, cannot be a source of good judgment and decision-making. | We appreciate the reviewer for their important concern about heterogeneity in clinical implications for opioids and physical therapy. Since our data does not contain specific diagnoses, procedures, or other clinical context, we have updated our limitations with: “Another limitation is the outcome of PCPV may not be generalizable to opioid prescriptions, real-world opioid utilization or misuse. Our data also does not distinguish between appropriate and inappropriate opioid prescriptions or physical therapy use, therefore our PCPV findings are limited in that they cannot be interpreted as appropriate opioid prescribing behavior. Since our data do not include opioid prescription volume or use, future research is needed to see if these results translate to opioid prescriptions or use and other low value treatments for musculoskeletal pain (e.g., surgery or injections).” |

**Reviewer #3**

| Reviewer comments | Author reply |
| --- | --- |
| The research question is important and methods are generally appropriate. However, several core issues need clarification before the manuscript is suitable for publication. | We thank the reviewer for helpful suggestions that have improved the quality of the manuscript. Below we address each specific comment by the reviewer. |
| PCPV outcome validity needs clarification (Approx. lines 102–110): PCPV reflects ARCOS pill shipments, not prescriptions or use. This limits interpretability. The authors should clearly justify why this metric is an appropriate proxy and strengthen the limitations. | We have clarified PCPV outcome in the methods with: “Our primary outcome was the per capita pill volume (PCPV) of opioids, defined as the annual per-capita shipments of oxycodone and hydrocodone pill shipment to retail pharmacies, calculated at the county-level in the ARCOS database”  We have also clarified that PCPV is not real-world prescription patterns in the limitations with: “Another limitation is the outcome of PCPV may not be generalizable to opioid prescriptions, real-world opioid utilization or misuse. Our data also does not distinguish between appropriate and inappropriate opioid prescriptions or physical therapy use, therefore our PCPV findings are limited in that they cannot be interpreted as appropriate opioid prescribing behavior. Since our data do not include opioid prescription volume or use, future research is needed to see if these results translate to opioid prescriptions or use and other low value treatments for musculoskeletal pain (e.g., surgery or injections).” |
| Unadjusted DID as the “primary” model is not convincing (Approx. lines 117–121): Given major state-level changes during 2006–2014 (Medicaid expansion, PDMP rollout, ACA), relying on unadjusted DID is questionable. Authors should either justify this more strongly or elevate adjusted analyses. | The unadjusted DID has strong parallel pre-trends that suggest that trends in the outcomes remained similar for 4 years before the treatment. Several sensitivity analyses were conducted including adjusted DID that further aligned with the primary analysis. Therefore, we have kept the primary analysis as the main analysis but have further added clarification between the primary and sensitivity analyses with: “Our sensitivity analyses including covariate-adjusted DID models, state-level clustering models, and synthetic control analyses for Hawaii were consistent with primary analysis results showing a reduction in PCPV after change in physical therapy direct access.” |
| Noise in ORD outcome (Approx. lines 168–177): ORD varies widely and is unstable at the county level. Mortality rates are highly noisy. Consider smoothing (e.g., 3-year averages) or acknowledge this more directly as a limitation. | We agree that ORD and mortality rates more broadly can be underpowered and thus have statistical uncertainty. We have added this to our limitations with: “Mortality rates can require large sample size to properly power analyses to identify statistically significant effects due to their rarity. ORD outcomes in our analysis have 95% confidence intervals that cannot rule out that the null effect is due to low power and not a lack of effect of physical therapy direct access policy on ORD.” |
| Exposure variable too simplified (Approx. lines 93–100): Direct access laws vary across states (limited, provisional, unrestricted). Treating exposure as purely binary may oversimplify policy differences. Clarify or justify. | We have clarified the treatment and control groups in our methods as suggested by the reviewer with: “Any other states were excluded due to having partial or provisional direct access prior to the study periods.”  Further, as the reviewer suggested, we have added to the limitations section with: “Finally, physical therapy direct access policies can be heterogenous (e.g., amount of physical therapy visits restricted) which may limit the generalizability of our findings.” |
| Synthetic control applied only to Hawaii is very limited (Approx. lines 124–131): Hawaii has unique demographic/geographic characteristics. Results from a single treated unit should not be generalized. Needs cautionary interpretation. | While Hawaii in our data was the most appropriate choice given being the single treated unit at a time, we agree with the reviewer that it can be hard to interpret given Hawaii’s unique characteristics. We have clarified this in the limitations with: “Our findings were robust to several different sensitivity analyses including using a different novel causal inference methodology synthetic control that has been used to assess policy change in other health conditions.^45^ Given Hawaii’s unique demographic characteristics, the synthetic control estimates provide the effect of physical therapy direct access policies for Hawaii which may not be generalizable to other states. However, given the alignment of the synthetic control results with the DID analyses, it provides further validity of the robustness of our findings.” |
| Missing discussion of co-occurring opioid policies: During this period, states implemented multiple opioid regulations (PDMP, pill mill laws, prescribing limits). These could confound effects unless addressed. At minimum, acknowledge as a limitation. | The reviewer makes an important point about other potential changes in policy that could have happened at the time of direct access to physical therapy policy change confounding our estimates. We have added this limitation with: “While the DID analysis with county and year fixed effects helps account for common temporal shocks, differential adoption of other opioid related policies (e.g., pill mill laws and prescribing limits) may bias our estimates.” |
