# Supplementary material for: The impact of physical therapy direct access policy on opioid shipments and opioid-related deaths: A difference-in-differences analysis
Source: PLoS One. 2026 Jun 10;21(6):e0333292. doi: 10.1371/journal.pone.0333292 (PMC13252735; doi:10.1371/journal.pone.0333292)
Supplement: Peer review history

# Peer review: Decision Letter 0

**DOI:** 10.1371/journal.pone.0333292.r001

**Authors:** Francesca Baratta

28 Jan 2026

PONE-D-25-49217

The Impact of Physical Therapy Direct Access Policy on Opioid Shipments and Opioid-related Deaths: A Difference-in-Differences Analysis

PLOS One

Dear Dr. Griffith,

Thank you for submitting your manuscript to PLOS ONE. After careful consideration, we feel that it has merit but does not fully meet PLOS ONE’s publication criteria as it currently stands. Therefore, we invite you to submit a revised version of the manuscript that addresses the points raised during the review process.

Please submit your revised manuscript by Mar 14 2026 11:59PM. If you will need more time than this to complete your revisions, please reply to this message or contact the journal office at plosone@plos.org. When you're ready to submit your revision, log on to https://www.editorialmanager.com/pone/ and select the 'Submissions Needing Revision' folder to locate your manuscript file.

Please include the following items when submitting your revised manuscript:

- A letter that responds to each point raised by the academic editor and reviewer(s). You should upload this letter as a separate file labeled 'Response to Reviewers'.
- A marked-up copy of your manuscript that highlights changes made to the original version. You should upload this as a separate file labeled 'Revised Manuscript with Track Changes'.
- An unmarked version of your revised paper without tracked changes. You should upload this as a separate file labeled 'Manuscript'.

If you would like to make changes to your financial disclosure, please include your updated statement in your cover letter. Guidelines for resubmitting your figure files are available below the reviewer comments at the end of this letter.

If applicable, we recommend that you deposit your laboratory protocols in protocols.io to enhance the reproducibility of your results. Protocols.io assigns your protocol its own identifier (DOI) so that it can be cited independently in the future. For instructions see: https://journals.plos.org/plosone/s/submission-guidelines#loc-laboratory-protocols. Additionally, PLOS ONE offers an option for publishing peer-reviewed Lab Protocol articles, which describe protocols hosted on protocols.io. Read more information on sharing protocols at https://plos.org/protocols?utm_medium=editorial-email&utm_source=authorletters&utm_campaign=protocols.

We look forward to receiving your revised manuscript.

Kind regards,

Francesca Baratta, PharmD, PhD

Academic Editor

PLOS One

Journal Requirements:

When submitting your revision, we need you to address these additional requirements.

1.Please ensure that your manuscript meets PLOS ONE's style requirements, including those for file naming. The PLOS ONE style templates can be found at

https://journals.plos.org/plosone/s/file?id=wjVg/PLOSOne_formatting_sample_main_body.pdf and

https://journals.plos.org/plosone/s/file?id=ba62/PLOSOne_formatting_sample_title_authors_affiliations.pdf

2. Please note that your Data Availability Statement is currently missing the repository name and/or the DOI/accession number of each dataset OR a direct link to access each database. If your manuscript is accepted for publication, you will be asked to provide these details on a very short timeline. We therefore suggest that you provide this information now, though we will not hold up the peer review process if you are unable.

3.If the reviewer comments include a recommendation to cite specific previously published works, please review and evaluate these publications to determine whether they are relevant and should be cited. There is no requirement to cite these works unless the editor has indicated otherwise.

[Note: HTML markup is below. Please do not edit.]

Reviewer's Responses to Questions

**Comments to the Author**

1. Is the manuscript technically sound, and do the data support the conclusions?

The manuscript must describe a technically sound piece of scientific research with data that supports the conclusions. Experiments must have been conducted rigorously, with appropriate controls, replication, and sample sizes. The conclusions must be drawn appropriately based on the data presented.

Reviewer #1: Yes

Reviewer #2: No

Reviewer #3: Yes

**********

2. Has the statistical analysis been performed appropriately and rigorously?

Reviewer #1: Yes

Reviewer #2: Yes

Reviewer #3: Yes

**********

3. Have the authors made all data underlying the findings in their manuscript fully available?

The PLOS Data policy requires authors to make all data underlying the findings described in their manuscript fully available without restriction, with rare exception (please refer to the Data Availability Statement in the manuscript PDF file). The data should be provided as part of the manuscript or its supporting information, or deposited to a public repository. For example, in addition to summary statistics, the data points behind means, medians and variance measures should be available. If there are restrictions on publicly sharing data—e.g. participant privacy or use of data from a third party—those must be specified.

Reviewer #1: Yes

Reviewer #2: Yes

Reviewer #3: No

**********

4. Is the manuscript presented in an intelligible fashion and written in standard English?

PLOS ONE does not copyedit accepted manuscripts, so the language in submitted articles must be clear, correct, and unambiguous. Any typographical or grammatical errors should be corrected at revision, so please note any specific errors here.

Reviewer #1: Yes

Reviewer #2: Yes

Reviewer #3: Yes

**********

5. Review Comments to the Author

Please use the space provided to explain your answers to the questions above. You may also include additional comments for the author, including concerns about dual publication, research ethics, or publication ethics. (Please upload your review as an attachment if it exceeds 20,000 characters)

Reviewer #1: (Suggestions for further Improvement)

Abstract

It is only wise that it expressly states that the policy exposure is measured at the state level, whereas the outcomes are measured at the county level. More so, a brief definition of the term per-capita volume of opioid pills in the form of annual per-capita shipments of oxycodone and hydrocodone pills to retail pharmacies should be given.

Introduction

The introduction of the knowledge gap can be made sharper; that is, one should add one or two sentences to the Introduction that will clearly indicate that no previous study has exploited inter-state variation in direct access physical therapy laws to measure the causal impact of the latter on the volumes of opioid pills shipped and opioid-related mortality.

Methods (Data sources & exposure)

1. The analysis clearly indicates that the treated and control states of county-year observations are clustered, with standard errors being clustered at the county level; sensitivity analyses are also done by clustering at the state level.

2. The selection of only Hawaii, Kansas, California, and Indiana as treated states was based on each one of them implementing the policy of direct-access in the study period and on similar demographic and economic factors; the eight never-treated states were chosen as a control group to achieve matching on these factors, and any states that implemented a partial access policy, or whose timing did not coincide, or which had incomplete data were excluded.

3. A brief description is provided of the heterogeneity of direct-access policies, which include free access to the policy, procedures with visitor time restrictions, etc.; this diversity can affect the scale of the estimated impacts and constrain the extrapolation of the policy findings.

Outcomes

ORD outcome is measured in 100,000 inhabitants per 100,000 inhabitants, but these values are not directly indicated in the manuscript as being age-adjusted or unadjusted. The latter methodological ambiguity should be tackled because the age distributions of different counties may differ significantly, thus affecting the comparability of the reported rates.

Statistical analysis

1. The Difference-in-Differences (DiD) model specification could be improved in terms of increased transparency, specifically in the clear definition of fixed-effects structures (e.g., state x year versus county x year) and the clear description of how time-varying covariates (including more than the usual controls of age, income, uninsurance, and dual eligibility) are operationalized (continuous or categorical).

2. A limitation of the study is that cross-state spillovers (like cross-border prescribing or mortality) may be present in the study. In addition, the synthetic-control approach is overly brevily treated, with more information given about how the pre-policy fit is assessed (as e.g. by root-mean-square prediction error), the use of either placebo-tests or permutation tests, and how the temporal window in which the synthetic control has been constructed and used is to be specified.

Results

1. The differences in the baselines in the treatment and control states, including high PCPV and ORD value, differences in income and coverage of insurance, are accepted but not discussed in terms of the possible impact on cause interpretation or external validity. It would therefore be wise to give a brief evaluation on whether the treated states are highly-burdened contexts.

2. The results are typically presented clearly, but the specifics that are of interest are only located in the appendix (e.g., synthetic control results, sensitivity analysis). In the primary Results section, it would be a good idea to summarise how these sensitivity estimates match (i.e. concordant or divergent) the major findings of DID.

3. In the case of ORD, the specified non-significant effect with the wide confidence interval that includes both substantive decreases and increases is reported, yet not placed in a relative change context or in a statistical power setting.

Discussion

1. The null effect of opioid-related mortality should be described in greater detail: the shortness of the follow-up, the research involving a high number of other mortality drivers, and the potential lack of statistical power in the form of county-level mortality results should be mentioned.

2. The impediments to PT access and the suggestion that the remaining states should emulate direct-access policies should be presented with more caution, clearly identifying those findings that are directly elucidated by this analysis as opposed to the conclusions made by the rest of the literature and the historical background of such policies.

Limitations (Additional limitations to consider)

The possibility of discrepancies between the distribution of the tablets with opioids and the trend of the real usage or intake of the prescription is real. In addition, the present study fails to involve data related to non-prescribed opioid use or poly-substance abuse.

References

It is seen that some of the web-based sources and Google Scholar entries, including the Washington Post database of ARCOS and the AHRF, are currently quoted by using only the path scholar.google.com and not by mentioning original reports or technical documents. To be on the safe side and assure verifiability in the long term, it would be wise to include direct quotes of the main sources.

Reviewer #2: A good study has been conducted to control the use of opioids, but due to the differences in the need for opioids in different types of diseases, the need for physical therapy, the existence of specific surgeries, etc., it has not been considered. As a result, this comparison, in my opinion, cannot be a source of good judgment and decision-making.

Reviewer #3: The research question is important and methods are generally appropriate. However, several core issues need clarification before the manuscript is suitable for publication.

1. PCPV outcome validity needs clarification (Approx. lines 102–110):

PCPV reflects ARCOS pill shipments, not prescriptions or use. This limits interpretability. The authors should clearly justify why this metric is an appropriate proxy and strengthen the limitations.

2. Unadjusted DID as the “primary” model is not convincing (Approx. lines 117–121):

Given major state-level changes during 2006–2014 (Medicaid expansion, PDMP rollout, ACA), relying on unadjusted DID is questionable. Authors should either justify this more strongly or elevate adjusted analyses.

3. Noise in ORD outcome (Approx. lines 168–177):

ORD varies widely and is unstable at the county level. Mortality rates are highly noisy. Consider smoothing (e.g., 3-year averages) or acknowledge this more directly as a limitation.

4. Exposure variable too simplified (Approx. lines 93–100):

Direct access laws vary across states (limited, provisional, unrestricted). Treating exposure as purely binary may oversimplify policy differences. Clarify or justify.

5. Synthetic control applied only to Hawaii is very limited (Approx. lines 124–131):

Hawaii has unique demographic/geographic characteristics. Results from a single treated unit should not be generalized. Needs cautionary interpretation.

6. Missing discussion of co-occurring opioid policies:

During this period, states implemented multiple opioid regulations (PDMP, pill mill laws, prescribing limits). These could confound effects unless addressed. At minimum, acknowledge as a limitation.

**********

6. PLOS authors have the option to publish the peer review history of their article (what does this mean?). If published, this will include your full peer review and any attached files.

If you choose “no”, your identity will remain anonymous but your review may still be made public.

**Do you want your identity to be public for this peer review?** For information about this choice, including consent withdrawal, please see our Privacy Policy.

Reviewer #1: **Yes:**MOHD ASIF

Reviewer #2: No

Reviewer #3: No

**********

[NOTE: If reviewer comments were submitted as an attachment file, they will be attached to this email and accessible via the submission site. Please log into your account, locate the manuscript record, and check for the action link "View Attachments". If this link does not appear, there are no attachment files.]

To ensure your figures meet our technical requirements, please review our figure guidelines: https://journals.plos.org/plosone/s/figures

You may also use PLOS’s free figure tool, NAAS, to help you prepare publication quality figures: https://journals.plos.org/plosone/s/figures#loc-tools-for-figure-preparation.

NAAS will assess whether your figures meet our technical requirements by comparing each figure against our figure specifications.

Attachment Submitted filename: Report-PONE-D-25-49217.docx

# Author comment: Author response to Decision Letter 1

**DOI:** 10.1371/journal.pone.0333292.r002

20 Mar 2026

Please see attachment.

Attachment Submitted filename: PLOS ONE Response Letter 2026 03 14.docx

# Peer review: Decision Letter 1

**DOI:** 10.1371/journal.pone.0333292.r003

**Authors:** Francesca Baratta

8 May 2026

The Impact of Physical Therapy Direct Access Policy on Opioid Shipments and Opioid-related Deaths: A Difference-in-Differences Analysis

PONE-D-25-49217R1

Dear Dr. Griffith,

We’re pleased to inform you that your manuscript has been judged scientifically suitable for publication and will be formally accepted for publication once it meets all outstanding technical requirements.

Within one week, you’ll receive an e-mail detailing the required amendments. When these have been addressed, you’ll receive a formal acceptance letter and your manuscript will be scheduled for publication.

An invoice will be generated when your article is formally accepted. Please note, if your institution has a publishing partnership with PLOS and your article meets the relevant criteria, all or part of your publication costs will be covered. Please make sure your user information is up-to-date by logging into Editorial Manager at Editorial Manager® and clicking the ‘Update My Information' link at the top of the page. For questions related to billing, please contact billing support.

If your institution or institutions have a press office, please notify them about your upcoming paper to help maximize its impact. If they’ll be preparing press materials, please inform our press team as soon as possible -- no later than 48 hours after receiving the formal acceptance. Your manuscript will remain under strict press embargo until 2 pm Eastern Time on the date of publication. For more information, please contact onepress@plos.org.

Kind regards,

Francesca Baratta, PharmD, PhD

Academic Editor

PLOS One

Reviewers' comments:

Reviewer's Responses to Questions

**Comments to the Author**

1. If the authors have adequately addressed your comments raised in a previous round of review and you feel that this manuscript is now acceptable for publication, you may indicate that here to bypass the “Comments to the Author” section, enter your conflict of interest statement in the “Confidential to Editor” section, and submit your "Accept" recommendation.

Reviewer #1: (No Response)

Reviewer #3: All comments have been addressed

**********

2. Is the manuscript technically sound, and do the data support the conclusions?

The manuscript must describe a technically sound piece of scientific research with data that supports the conclusions. Experiments must have been conducted rigorously, with appropriate controls, replication, and sample sizes. The conclusions must be drawn appropriately based on the data presented.

Reviewer #1: Yes

Reviewer #3: Yes

**********

3. Has the statistical analysis been performed appropriately and rigorously?

Reviewer #1: Yes

Reviewer #3: Yes

**********

4. Have the authors made all data underlying the findings in their manuscript fully available?

The PLOS Data policy requires authors to make all data underlying the findings described in their manuscript fully available without restriction, with rare exception (please refer to the Data Availability Statement in the manuscript PDF file). The data should be provided as part of the manuscript or its supporting information, or deposited to a public repository. For example, in addition to summary statistics, the data points behind means, medians and variance measures should be available. If there are restrictions on publicly sharing data—e.g. participant privacy or use of data from a third party—those must be specified.

Reviewer #1: Yes

Reviewer #3: Yes

**********

5. Is the manuscript presented in an intelligible fashion and written in standard English?

PLOS ONE does not copyedit accepted manuscripts, so the language in submitted articles must be clear, correct, and unambiguous. Any typographical or grammatical errors should be corrected at revision, so please note any specific errors here.

Reviewer #1: Yes

Reviewer #3: Yes

**********

6. Review Comments to the Author

Please use the space provided to explain your answers to the questions above. You may also include additional comments for the author, including concerns about dual publication, research ethics, or publication ethics. (Please upload your review as an attachment if it exceeds 20,000 characters)

Reviewer #1: (No Response)

Reviewer #3: The authors have adequately addressed all previous reviewer comments and concerns. The revisions have improved the clarity and overall quality of the manuscript, and I have no further major concerns. I recommend acceptance.

**********

7. PLOS authors have the option to publish the peer review history of their article (what does this mean?). If published, this will include your full peer review and any attached files.

If you choose “no”, your identity will remain anonymous but your review may still be made public.

**Do you want your identity to be public for this peer review?** For information about this choice, including consent withdrawal, please see our Privacy Policy.

Reviewer #1: **Yes:**Mohd Asif

Reviewer #3: No

**********

# Editor report: Acceptance letter

**DOI:** 10.1371/journal.pone.0333292.r004

**Authors:** Francesca Baratta

PONE-D-25-49217R1

PLOS One

Dear Dr. Griffith,

I'm pleased to inform you that your manuscript has been deemed suitable for publication in PLOS One. Congratulations! Your manuscript is now being handed over to our production team.

At this stage, our production department will prepare your paper for publication. This includes ensuring the following:

* All references, tables, and figures are properly cited

* All relevant supporting information is included in the manuscript submission,

* There are no issues that prevent the paper from being properly typeset

You will receive further instructions from the production team, including instructions on how to review your proof when it is ready. Please keep in mind that we are working through a large volume of accepted articles, so please give us a few days to review your paper and let you know the next and final steps.

Lastly, if your institution or institutions have a press office, please let them know about your upcoming paper now to help maximize its impact. If they'll be preparing press materials, please inform our press team within the next 48 hours. Your manuscript will remain under strict press embargo until 2 pm Eastern Time on the date of publication. For more information, please contact onepress@plos.org.

You will receive an invoice from PLOS for your publication fee after your manuscript has reached the completed accept phase. If you receive an email requesting payment before acceptance or for any other service, this may be a phishing scheme. Learn how to identify phishing emails and protect your accounts at https://explore.plos.org/phishing.

If we can help with anything else, please email us at customercare@plos.org.

Thank you for submitting your work to PLOS ONE and supporting open access.

Kind regards,

PLOS ONE Editorial Office Staff

on behalf of

Dr. Francesca Baratta

Academic Editor

PLOS One
